# Sodium Intake During an Ultramarathon Does Not Prevent Muscle Cramping, Dehydration, Hyponatremia, or Nausea

**DOI:** 10.1186/s40798-015-0040-x

**Published:** 2015-12-22

**Authors:** Martin D. Hoffman, Kristin J. Stuempfle, Taylor Valentino

**Affiliations:** 1 Department of Physical Medicine & Rehabilitation, Department of Veterans Affairs, Sacramento VA Medical Center, Northern California Health Care System, 10535 Hospital Way, Sacramento, CA 95655-1200 USA; 2 Department of Physical Medicine & Rehabilitation, University of California Davis Medical Center, Sacramento, CA USA; 3 Health Sciences Department, Gettysburg College, Gettysburg, PA USA; 4 Department of Kinesiology, San Francisco State University, San Francisco, CA USA

## Abstract

**Background:**

Ultramarathon runners commonly believe that sodium replacement is important for prevention of muscle cramping, dehydration, hyponatremia, and nausea during prolonged continuous exercise. The purpose of this study was to measure total sodium intake to determine if these beliefs are supported.

**Methods:**

Participants of a 161-km ultramarathon (air temperature reaching 39 °C) provided full dietary information during the race, underwent body weight measurements before and after the race, completed a post-race questionnaire about muscle cramping and nausea or vomiting during the race, and had post-race plasma sodium concentration measured.

**Results:**

Among 20 finishers providing dietary data, mean (±SD) total sodium intake was 13,651 ± 8444 mg (range 2541–38,338 mg), and sodium in food and drink accounted for 66 % of the sodium when averaged across subjects (range 34–100 %). Sodium intake rates were similar when comparing the 10 % of subjects who were hyponatremic with those who were not hyponatremic, the 39 % with muscle cramping or near cramping with those without cramping, and the 57 % who reported having symptoms of nausea or vomiting with those without these symptoms. Weight change between race start and finish was significantly related to rate of sodium intake (*r* = 0.49, *p* = 0.030) and total sodium intake (*r* = 0.53, *p* = 0.016), but the maximum weight loss among those taking the least total sodium (<4400 mg total sodium during the race) was 4–5 % below the weight measured immediately pre-race.

**Conclusions:**

Exercise-associated muscle cramping, dehydration, hyponatremia, and nausea or vomiting during exercise up to 30 h in hot environments are unrelated to total sodium intake, despite a common belief among ultramarathon runners that sodium is important for the prevention of these problems.

## Key Points

- Even though most study participants (93.3 %) used electrolyte capsules during the race, food and drink were generally the sources for most sodium consumed by participants of this 161-km ultramarathon.
- Study participants averaged over 9 times the adequate daily sodium intake during the race, and one individual took in over 25 times the adequate daily intake.
- Opposed to common belief among ultramarathon runners, total sodium intake does not appear to be important in preventing muscle cramping, nausea or vomiting, hyponatremia, and dehydration during a running race in hot conditions lasting up to 30 h.

## Background

Ultramarathon runners have a common belief that sodium intake during exercise prevents muscle cramping, dehydration, and hyponatremia [1]. There is also a conventional perception that fluid or electrolyte imbalances are a cause of nausea, a common symptom during ultramarathons [2–7], and that sodium intake may prevent such symptoms [8]. For these reasons, most ultramarathon runners use sodium supplements during competitions. In fact, prior research at the 161.3-km Western States Endurance Run (WSER) demonstrated that 90–96 % of runners used sodium supplements during the event [9, 10]. Despite the general acceptance that there are such benefits from sodium intake during ultramarathons, there has been little investigation to support or challenge these ideas.

In a study at the 2013 WSER in which nearby ambient temperatures reached 39 °C, we found that runners could maintain proper hydration without using sodium supplements and by drinking to thirst [9]. At the 2014 WSER, we extended these findings to demonstrate that supplemental sodium enhanced body weight maintenance, but those who were not using sodium supplements maintained a more appropriate hydration level than those consistently using sodium supplements [11]. We also found that the use of sodium supplements and intake rate of sodium in supplements did not differ between those with and without muscle cramping [12] and that inadequate sodium supplementation was not a cause for the development of hyponatremia [1]. Furthermore, the rate of sodium intake in supplements and in buffered sodium supplements was found to be unrelated to symptoms of nausea or vomiting [13].

This prior work suggests that sodium supplementation during exercise up to 30 h is not important for the prevention of muscle cramping, dehydration, hyponatremia, and nausea or vomiting. However, this work was limited by focusing on supplemental sodium intake without examination of total sodium intake. Collection of the required information to determine total sodium intake is not feasible with a large cohort of runners, but in the present work, we report the findings on a subset of runners who provided full dietary data during the 2014 WSER. This allows for an examination of the importance of total sodium intake at the prevention of muscle cramping, dehydration, hyponatremia, and nausea or vomiting during the race.

## Methods

The study was performed at the 2014 WSER. The WSER is a 161.3-km ultramarathon through the Sierra Nevada Mountains of northern California. The course has 5500 m of cumulative climb and 7000 m of cumulative descent. Other details of the race have been provided previously [4, 14–16]. Nearby weather station ambient temperatures during the race ranged from a low 0 °C shortly after the start to a high 31.7 °C in the afternoon, which was close to the historical median high temperature for this event. However, we measured (Vantage Vue Wireless Weather Station, Davis Instruments, Vernon Hills, IL, USA) a maximum on-course air temperature of 39 °C at which time the relative humidity was 13 %. There were 24 aid stations on the course that were stocked with various foods, fluids, and electrolyte capsules.

This study received approval from our institutional review boards with electronic consent obtained from those participating in a post-race questionnaire and formal signed consent obtained from those participating in the dietary analysis. Recruitment for participants to provide dietary analyses took place by pre-race electronic correspondence with all race entries. Pre-race electronic correspondence also alerted race participants that they would be requested to complete a post-race web-based questionnaire. All race entries were eligible to participate in the study.

Body mass was measured to the nearest 0.1 kg on all race participants within 1.5 h before the start of the race and when each reached 47.8 km, 89.6 km, 125.5 km, and the finish using calibrated scales (Health O Meter, model 349KLX, Boca Raton, FL, USA) placed on firm, level surfaces. During each measurement, the runner was clothed in running clothes and shoes, but other items such as waist packs and hydration vests were removed, and nothing was permitted in the runner’s hands. Prior to the event, the scales were examined for consistency, and correction equations were developed to standardize all weight measurements to a single scale as done previously [9].

Race finishers were invited to provide a blood sample within a few minutes after finishing the race. For those runners willing to provide the sample, blood was drawn while they were seated into heparinized tubes via an antecubital vein and stored in a cooler until analyzed for plasma sodium concentration by a clinical laboratory (Siemens Aktiengesellschaft, Dimension EXL, Munich, Germany).

An electronic invitation to complete a post-race questionnaire was sent to all starters during the event. Reminder emails were distributed to runners who had not completed the survey 7 and 12 days later, and the survey was closed 15 days after the race. The questionnaire requested information on the presence or absence of nausea or vomiting during each of four segments of the race defined by the sites where body weights were measured. The questionnaire also asked about whether or not the runner had experienced muscle cramping during each of the four race segments. Answer options included yes, no, and “almost, but was able to control any full-blown cramping” (subsequently referred to as “near cramping”). Near cramping was included as an answer option because runners sometimes sense impending muscle cramping that can be aborted with various interventions, such as altering gait pattern or stretching.

Dietary analysis used a multipronged approach previously used at this event with built-in redundancy to maximize accurate accounting of food, fluid, and electrolyte capsule intake [6, 17]. Approximately 1 week pre-race, via electronic correspondence, subjects provided a plan of the food, fluid, and electrolyte capsules they intended to consume during the race. Each subject was then interviewed about their plan during race registration to clarify any questions about the plan and to familiarize them with the required detail about brand, flavor, and amount of each consumed item. Volumes of bottles and hydration pack bladders were recorded at that time. During the race at each site where body weights were obtained, subjects provided details on food, fluid, and electrolyte capsule intake during that race segment and food wrappers were collected. Approximately 1 week post-race, the subjects were provided their race diet logs via electronic correspondence to review for completeness and accuracy. Nutritionist Pro (Axxya Systems, Stafford, TX, USA) software was then used to analyze the nutritional composition of the foods, fluids, and electrolyte capsules consumed. Nutritional information for items not included in Nutritionist Pro was obtained from the manufacturer and added to the software database. Rate of sodium intake was determined using official split and finish times.

Two-group comparisons of continuous data were made with the unpaired *t* test or Mann-Whitney test depending on whether or not the data passed normality testing with the D’Agostino-Pearson test. Between-group comparisons of categorical data were made with the Fisher’s exact test. Correlations between two variables were determined with Pearson’s correlation analyses. Statistical significance was set at *p* < 0.05.

## Results

Of the 376 race starters, 30 race participants enrolled in the dietary analysis portion of the study, of which 20 finished the race, 23 completed the post-race survey, and 18 did both. Of those completing the survey, 65 % did so within 7 days of the end of the race and 96 % did so within 10 days. All 20 finishers providing dietary records also provided a post-race blood sample. Their mean (±SD) finish time was 26.83 ± 2.39 h, which was slower (*p* = 0.023) than the 24.83 ± 3.79 h for the full group of 296 race finishers. However, the age of this study group (43 ± 10 years) was similar (*p* = 0.37) to the age of the full group of race finishers (41 ± 9 years), and a similar proportion (*p* = 0.55) were women for the study group and the full group of finishers (25 vs. 19 %).

Among the 20 finishers providing dietary records, the mean (±SD) total sodium intake was 13,651 ± 8444 mg (range 2541–38,338 mg). Sodium consumed in food and drink amounted to 8434 ± 4183 mg, and sodium intake from electrolyte capsules was 5217 ± 5614 mg, so when averaged across subjects, 66 % (range 34–100 %) of the sodium came from food and drink. Considering all 30 runners providing dietary records, 28 (93 %) used electrolyte capsules during the race, and the mean (±SD) rate of sodium intake was 438 ± 280 mg/h with the sodium from food and drink accounting for an average of 60 % (range 13–100 %) of total sodium intake.

Of the 20 finishers providing dietary records, 2 (10 %) were hyponatremic at the finish. Both were asymptomatic and had plasma sodium concentrations of 130 and 134 mmol/L. They had total sodium intakes of 8727 and 12,723 mg and rates of sodium intake of 297 and 554 mg/h, respectively. These values were comparable (*p* = 0.62 and 0.70) to the mean (±SD) total (13,976 ± 8838 mg) and rate (515 ± 315 mg/h) of sodium intake among those finishers who were not hyponatremic. Additionally, there was no relationship for post-race plasma sodium concentration with total sodium intake across the entire race (*p* = 0.35), rate of sodium intake across the entire race (*p* = 0.33), total sodium intake across the last race segment (*p* = 0.69), or rate of sodium intake across the last race segment (*p* = 0.61) among the 20 finishers providing dietary records.

**Fig. 1:**
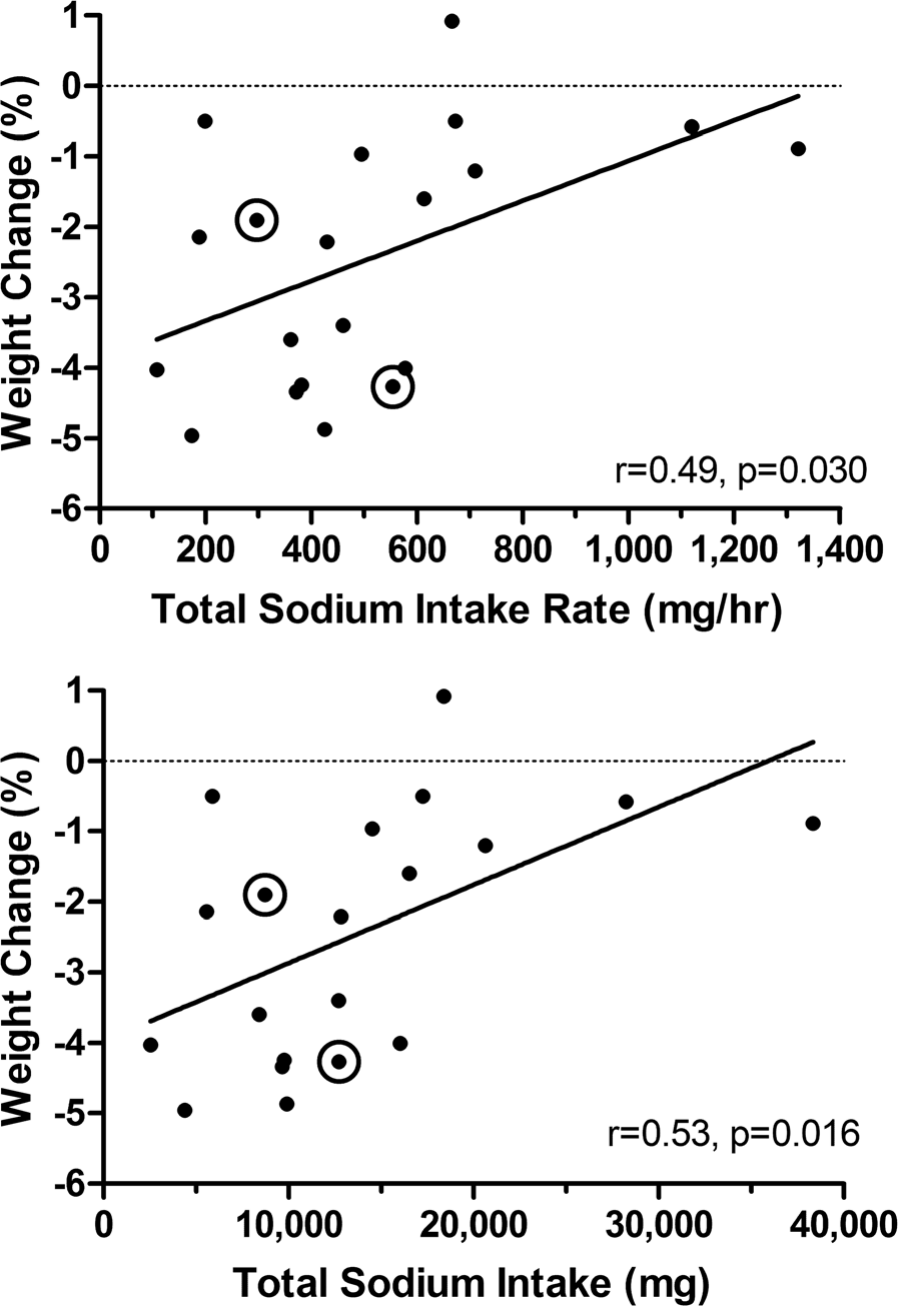
Relationship of percentage change in body weight from immediately before the race start to the finish with rate of sodium intake (*top*) and total sodium intake (*bottom*) for the 20 race finishers participating in the dietary analysis. The *open circles* distinguish those finishing with hyponatremia. The subject with the lowest plasma sodium concentration had the lower sodium intake

The relationship of weight change between race start and finish with sodium intake among finishers is shown in Fig. 1. Whether considering rate of sodium intake (*r* = 0.49, *p* = 0.030) or total sodium intake (*r* = 0.53, *p* = 0.016), the relationships were significant.

**Fig. 2:**
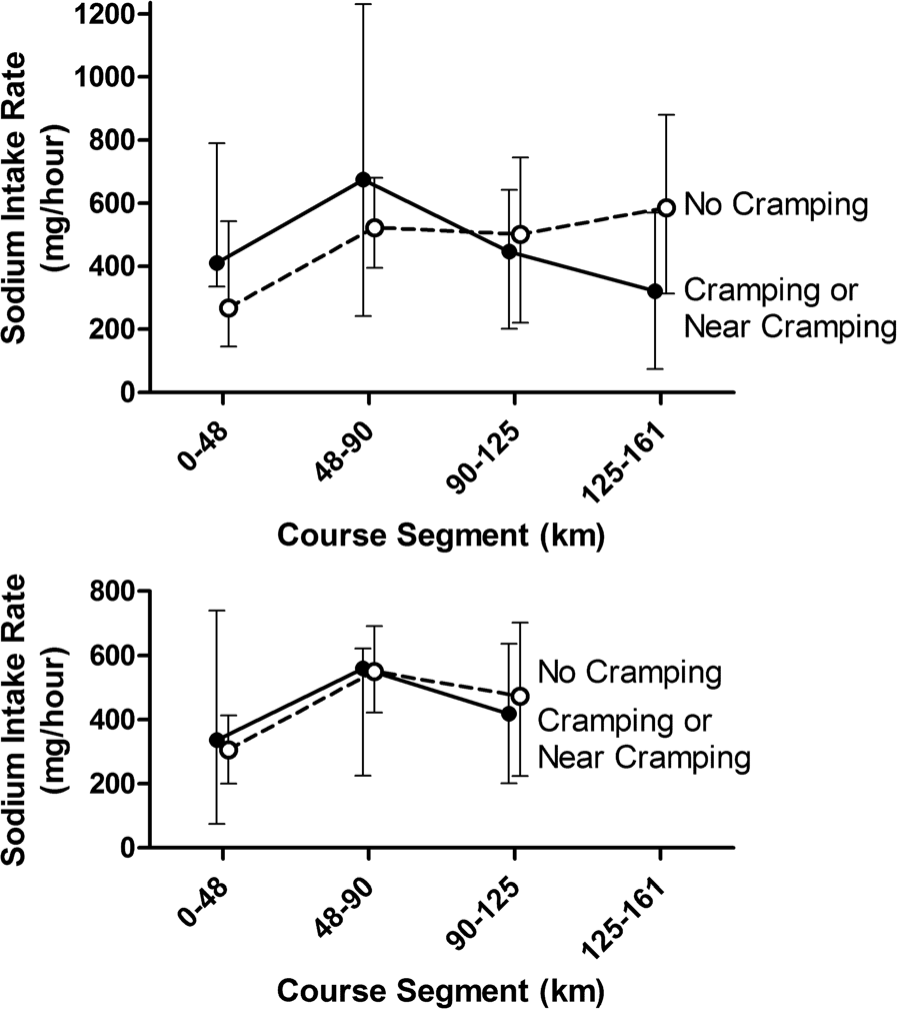
Median sodium intake rate during each course segment for those with cramping or near cramping and those without cramping during the same segment as the symptoms (*upper graph*) and during the segment before the symptoms occurred (*lower graph*). Data include finishers and non-finishers. *Error brackets* represent interquartile range. Data points are offset slightly along the horizontal axis for clarity of the error brackets. There were no group differences

Among the 23 study participants who completed the post-race survey, 9 (39 %) had muscle cramping or near cramping in some segment, and 14 had no cramping in any segment. Sodium intake rate was not different between those with and without cramping whether comparing intake during the course segment where cramping or near cramping occurred or the intake during the prior segment (Fig. 2).

**Fig. 3:**
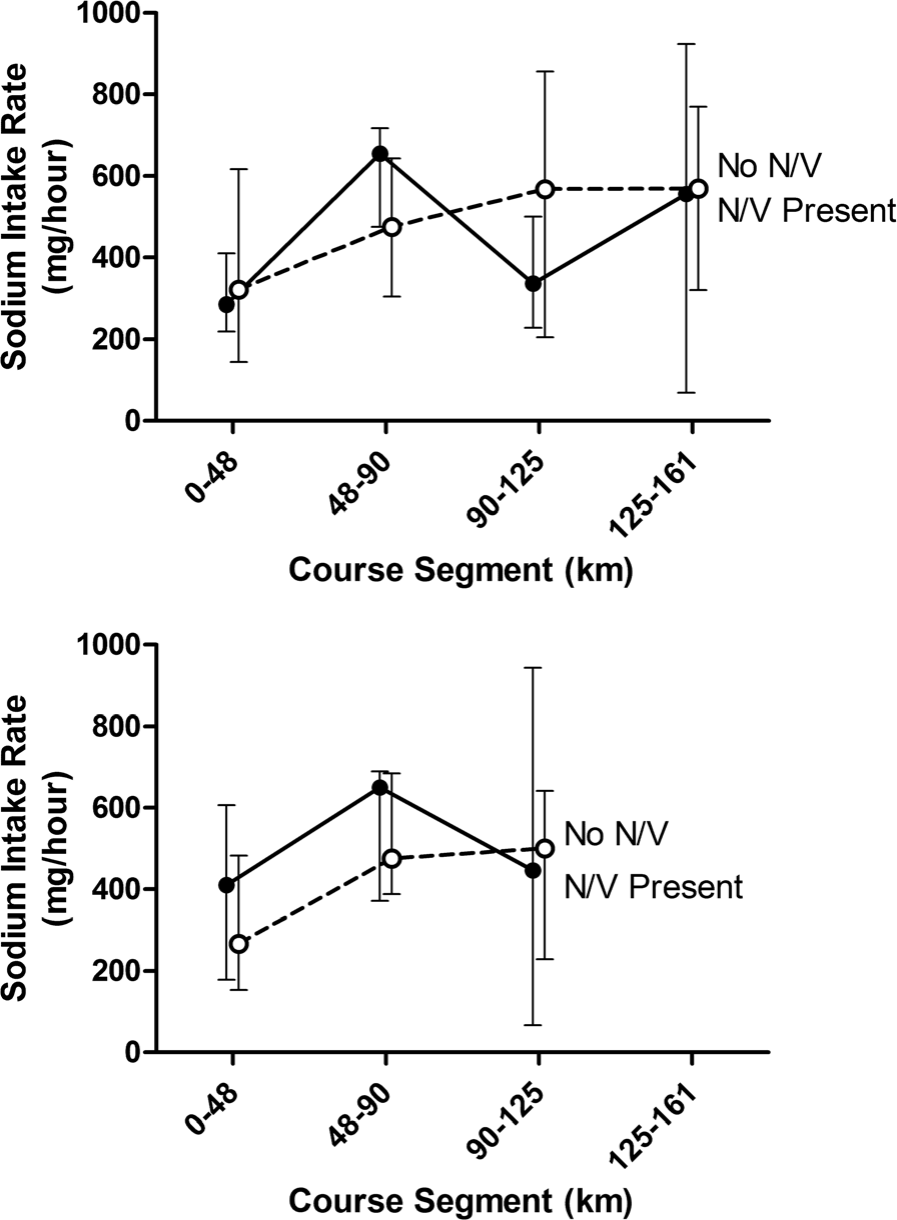
Median sodium intake rate during each course segment for those with and without nausea or vomiting (N/V) during the same segment as the symptoms (*upper graph*) and during the segment before the symptoms occurred (*lower graph*). Data include finishers and non-finishers. *Error brackets* represent interquartile range. Data points are offset slightly along the horizontal axis for clarity of the error brackets. There were no group differences

There were 13 runners (57 %) who reported having symptoms of nausea or vomiting during the race among the 23 who completed the post-race survey. Whether comparing intake during the course segment where nausea or vomiting occurred or the intake during the prior segment, sodium intake rate was not different between those with and without nausea or vomiting (Fig. 3).

## Discussion

The key findings of this work are that total sodium intake was not related to the presence of muscle cramping and nausea or vomiting and that low total sodium intake was not linked with hyponatremia or inadequate maintenance of hydration. This implies that total sodium intake was not important in preventing muscle cramping, nausea or vomiting, hyponatremia, or dehydration during a running race in hot conditions lasting up to 30 h. These findings support accumulating evidence that sodium intake is not as valuable during prolonged exercise as has been conventionally believed.

Mean sodium intake among the study participants finishing the race was 13,651 mg, but one subject took in over 38,000 mg during the race. While in general, food and drink were the sources for most sodium (average >60 % among subjects), there was considerable variability among subjects (range 34–100 % among finishers). For perspective, a daily sodium intake of 1500 mg is considered adequate, and it is recommended that sodium intake be limited to less than 2300 mg per day [18]. This means that our study population averaged over 9 times the adequate daily intake during the race, and one individual took in over 25 times the adequate daily intake. It also seems that this population may take in greater sodium than has been reported in other ultramarathon running competitions. For instance, data suggesting an average total sodium intake rate of 195 mg/h during a five-stage ultramarathon event has been reported [19], which seems considerably less than the mean rate of sodium intake of 438 mg/h among our subjects. However, the running duration in our study was much longer than that of the other work with the longest stage averaging less than 8 h. We presume that such high sodium intakes in our study population are due to a combination of salt craving, high availability of salty foods at the event, and the common belief among ultramarathon runners that ongoing replacement of sodium lost during the event through sweating is important for preventing muscle cramping, dehydration, hyponatremia, and nausea or vomiting, issues that are legitimate concerns among individuals participating in an ultramarathon.

We have previously reported data on supplemental sodium intake from the same race that was examined in the present work. Those reports demonstrated there to be no difference in supplemental sodium intake rate between those with and without muscle cramping [12], those with and without nausea or vomiting [13], and those with and without post-race hyponatremia [1]. That work was important in examination of the potential benefit of sodium supplementation, but it was limited in that total sodium intake was not reported because such information is not feasibly acquired on a large sample. The present work augments those prior reports with information on total sodium intake for a subset of runners, which in combination yields evidence that neither supplemental sodium intake nor total sodium intake relate to muscle cramping, nausea or vomiting, and post-race hyponatremia.

Prior work also demonstrated that, while supplemental sodium enhanced body weight maintenance, those who were not using sodium supplements maintained a more appropriate weight than those consistently using sodium supplements [9–11]. The present work similarly demonstrates the presence of a relationship between percentage weight change and total sodium intake. More importantly though is the finding that the maximum weight loss among those taking the least total sodium (<4400 mg total sodium during the race) was 4–5 % from weight immediately pre-race. This amount of weight loss is considered to be in the appropriate range for avoidance of performance-altering dehydration during an event of this nature, given the extent of expected utilization of stored glycogen and fat [20].

While the sample size for the present work was relatively small, we have evidence that it was reasonably representative of the larger group of runners. Participants of the present study ran an average of 2 h slower than the full group of race finishers, which it not unusual for those willing to participate in research during a competition. But, the age and sex distributions were similar between the present study group and the full group of runners. Furthermore, the incidence of muscle cramping or near cramping (39 vs. 41 %), nausea or vomiting (57 vs. 60 %), and post-race hyponatremia (10 vs. 7 %) was quite similar between the present sample and the larger study group. We also acknowledge that some of our conclusions are based on a small number of subjects with hyponatremia and that both hyponatremic runners were only mildly hyponatremic and asymptomatic. Furthermore, since we did not perform pre-race blood testing, we cannot assure that all runners were normonatremic at the start of the race, though of the 62 runners undergoing pre-race blood sampling at other 161-km ultramarathons where we have done investigations [7, 21], all have been normonatremic. Despite these potential limitations, the findings demonstrate that large amounts of sodium are not required to complete a 161-km ultramarathon without developing hyponatremia.

## Conclusions

During continuous exercise up to 30 h in hot environments, sodium does not protect against muscle cramping and nausea or vomiting nor are large amounts of sodium necessary to avoid hyponatremia or dehydration.
